# Palmatine treats urticaria by reducing inflammation and increasing autophagy

**DOI:** 10.3389/fimmu.2023.1268467

**Published:** 2023-11-14

**Authors:** Tian Xiao, Xingzhi Yu, Liping Yang, Xiaohua Duan

**Affiliations:** Yunnan Key Laboratory of Dai and Yi Medicines, Yunnan University of Chinese Medicine, Kunming, Yunnan, China

**Keywords:** chronic spontaneous urticaria, palmatine, inflammation, autophagy, ethnic medicine

## Abstract

**Introduction:**

Chronic spontaneous urticaria (CSU) is mainly manifested as wheals and erythema on the skin accompanied by itching, which will cause emotional anxiety and seriously affect the quality of life in patients. Palmatine (PAL) is a main chemical component of Yajieshaba, which has been found to effectively alleviate the symptoms of food allergy. However, its role and mechanism in CSU remain unclear. The present study aimed to investigate the protective effect of PAL on CSU rats.

**Methods:**

We replicated the CSU rat model by intraperitoneal injection of ovalbumin (OVA) in rats on days 0, 2, 4, and 14, with a double dose given on the last challenge. PAL, loratadine and saline were given by gavage from day 5 to day 14. We observed the skin pathologic changes, mast cell degranulation, immune factor levels, inflammatory response and autophagy-related protein expression in CSU rats.

**Results:**

We found PAL treatment to be effective in alleviating CSU-like skin lesions and reducing itching and mast cell degranulation in rats. Compared with the OVA group, the levels of immune and inflammatory factors were significantly reduced, neutrophil recruitment was alleviated, suggesting a reduced inflammatory response. The autophagy results showed that PAL further increased the expression of LC3, Beclin-1 and p-LKB1, p-AMPK, Atg5, Atg12 and Atg5-Atg12, while P62 and p-p70S6K1 expression decreased. They collectively suggested that autophagic flux was activated after PAL treatment. However, there was an increase in the expression of LC3I, probably due to the fact that PAL induced its accumulation in order to provide substrate for the generation of more LC3II.

**Discussion:**

Overall, PAL had a protective effect on CSU in normal rats, activated the expression of autophagy and improved the inflammatory response.

## 1 Introduction

Urticaria is a frequent allergic skin disease that occurs in the spring (1). The prevalence of urticaria in adults is about 15-23%, with the cause being undetermined in 70% of these patients (2). Chronic spontaneous urticaria (CSU) is defined as the unprovoked appearance of wheals, erythema, and severe skin pruritus, lasting less than 24 hours (as opposed to urticarial vasculitis) and relapsing for more than 6 weeks (3, 4). Patient anxiety is aggravated by itching and facial changes during the acute phase, with this anxiety in turn exacerbating the characteristics of CSU, thereby seriously affecting patient quality of life (5). Failure to intervene in a timely and effective manner is also associated with further exacerbation of damage to the patient’s immune system, which can be life-threatening in severe cases (6). The extensive use of antihistamines and/or glucocorticoids to treat CSU can result in neurological adverse effects, such as recurrent disease, dependence, dizziness and drowsiness (7). Therefore, it is urgent to treat the symptoms of patients and improve their quality of life.

Clinically, CSU is characterized as an immunoglobulin E (IgE) antibody-mediated type I allergic reaction (8). Stimulants in the external environment can trigger the production of IgE, which binds to mast cells and eosinophils (9), activating mast cell degranulation and stimulating eosinophils, which release inflammatory mediators such as histamines and cytokines, resulting in a sensitized state (10). These cytokines and other mediators induce capillary dilation, alter permeability, and stimulate the infiltration of tissue fluid into the dermis, resulting in edema and the formation of bright wheals (11). Simultaneously, these mediators will irritate the peripheral nerves, causing symptoms such as pruritus (12). Thus, altering the properties of mast cells and reducing inflammation remain key steps in the treatment of CSU.

Skin is extremely poor in nutrient resources (13). Autophagy recycles damaged cells at the onset of skin diseases through several steps, including vesicle formation, membrane elongation and fusion, lysosomal degradation, and recycling (14). Normal cells are supplied with energy during this process of renewal, maintaining homeostasis of the skin environment (15). Autophagy also plays a crucial role in selectively inhibiting IgE-induced mast cell degranulation and is a key target for the treatment of allergic diseases (16). Activation of autophagy has also been shown to drastically reduce inflammatory vesicle activity and inhibit the secretion of pro-inflammatory signals by immune cells (17), thus reducing inflammation caused by allergies (18). Pruritus in damaged skin has been shown to result from stimulation of the neuronal system by inflammatory mediators released by immune cells, with the induction of autophagy found to significantly alleviate this cascade of reactions (19). Paeoniflorin was shown to accelerate the elimination of CSU inflammatory responses and effectively improve the pathological symptoms of CSU by activating the LKB1/AMPK autophagy pathway (20). Overall, these findings indicate that autophagy plays an important role in CSU.

New medicines have been derived from traditional or ethnic medicines worldwide (21). Dai medicine, as one of the four major ethnic medicines in Chinese traditional medicine, has a long history and is a valuable wealth summarized and formed by the Dai people through long-term exploration and practice (22). Yajieshaba is one of the classic prescriptions of Dai medicine that has been developed into hospital preparations (23). Yajieshaba is widely used to treat diseases triggered by allergies, such as food allergies and intestinal inflammation (24). Palmatine (PAL), one of the main active ingredients of Yajieshaba, has been found to significantly reduce inflammatory factors in the sera of ovalbumin (OVA)-sensitized mice and to effectively alleviate the symptoms of food allergy (25). In addition, an herbal formulation developed to treat food allergies was found to counteract allergic reactions to peanuts by attenuating IgE-mediated degranulation of RBL-2H3 and human skin mast cells, and PAL was found to significantly inhibit the degranulation of RBL-2H3 cells (26). Interestingly, PAL has been found to exert significant anti-inflammatory effects in a wide range of diseases, including kidney inflammation, liver inflammation, osteoarthritic inflammation, pneumonia, and mucosal inflammation (27–31). PAL treatment was found to significantly enhance the transformation of LC3-II, the degradation of p62 and the expression of the autophagic proteins ATG5 and ATG7 in a cell model of alcoholic liver injury, thereby inhibiting hepatocyte apoptosis (32). Of interest, Electrospun nanofibrous scaffolds, PCL/GE/PALs, developed as excipients for loading PAL, were found to contribute to the sustained release of PAL at skin lesions, and this would suggest the possibility of topical administration of PAL (33). These findings suggested that PAL may be effective in the treatment of CSU.

The present study evaluated the immunomodulatory activities of PAL in a rat model of OVA-induced CSU. The effects of PAL on CSU-associated pathologic changes in skin and inflammatory factors were investigated. The potential mechanisms of action of PAL were explored, such as whether its activity involved the induction of autophagy. These findings may provide a compelling rationale for the clinical application of Yajieshaba and PAL in the treatment of CSU.

## 2 Material and methods

### 2.1 Animals

Twenty-four male Sprague-Dawley (SD) rats, aged 8 weeks and weighing about 20 g, were obtained from Changzhou Cavens Laboratory Animal Co. Ltd. (Animal Qualification Certificate No.: SCXK (Su) 2022-0010). The rats were maintained at a temperature of 18-25°C and a relative humidity of 40-60%, with a 12 h light/dark cycle and free access to food and water. All animal experiments were approved by the Professional Committee for animal ethics of Yunnan University of Chinese Medicine (Approval No. R-062022157). The experimental animals were cared for and used according to the guidelines of the American National Institute of Health, with every effort made to minimize animal numbers and suffering.

### 2.2 Experimental schedule and model replication

**Figure 1:**
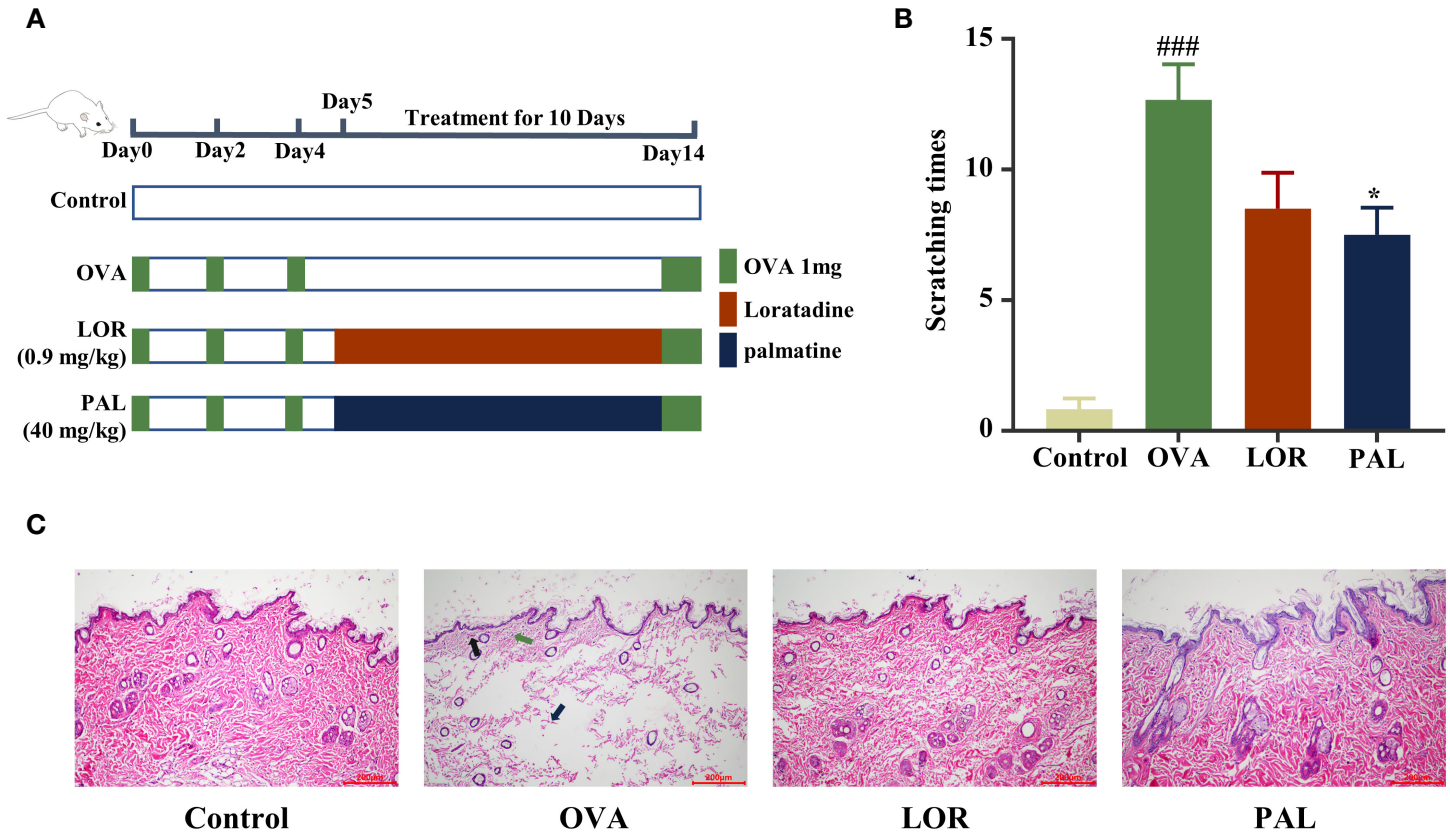
Experimental protocol **(A)**. The effects of PAL on scratching behavior **(B)** and skin histopathology **(C)**. Black arrows: inflammatory cell infiltration. Green arrow: capillary dilation. Blue arrows: collagen fiber bundles broken and lightened. Data are presented as mean ± standard deviation (SD). ^###^P<0.001 vs. Control group. ^*^P<0.05 vs. OVA group. OVA, ovalbumin; LOR, loratadine; PAL, palmatine.

After one week of acclimatization, the rats were randomly divided into four groups of six rats each. Rats in the OVA, OVA+loratadine (LOR) and OVA+PAL groups were intraperitoneally injected with an alum suspension containing 1 mg OVA (S7951, Sigma Aldrich, St. Louis, MO, USA) on days 0, 2, and 4 and with an alum suspension containing 2 mg OVA on day 14. Rats in the OVA, OVA+LOR, and OVA+PAL groups were intragastrically injected with saline, 0.9 mg/kg LOR (HY-17043, MCE, Shanghai, China), and 40 mg/kg PAL (AFBF2751, ABPHYTO, Chengdu, China), respectively, once a day on days 5-14. Rats in the Control group were intraperitoneally and intragastrically injected with saline according to the above schedule (**Figure 1A**). LOR, a second-generation histamine, was chosen as a positive control because it is used in the first-line treatment of CSU (34).

### 2.3 Blood and tissue samples

Orbital blood was obtained from each rat 1 h after the last administration of drug, and the blood samples were placed in PU tubes. After blood sampling, the rats were euthanized by cervical dislocation specified in the AVMA Guidelines for the Euthanasia of Animals: 2020 Edition. Skin tissue samples were obtained from the shaved back of each rat. During the study period, none of these animals developed humane endpoint indications, such as non minus;feeding, dyspnea, convulsion or hypothermia, or died prematurely.

### 2.4 Endpoint indicators

#### 2.4.1 Scratching behavior

Beginning 10 min after the last injection of OVA, rat scratching behavior was monitored for 20 min. Indicators of scratching included scratching the head with a front paw, scratching the trunk with a back paw, and mouthing all parts of the body.

#### 2.4.2 Hematoxylin-eosin staining

The skin tissue samples were fixed overnight in 4% paraformaldehyde at 4°C, dehydrated using a gradient of low to high concentrations of ethanol, and soaked in xylene twice for 1 h each. Tissue samples were embedded in paraffin and cut into 5 μm thick sections. The paraffin sections were baked in an electrically heated oven at 60°C for 3 h, deparaffinized with xylene, dewaxed using a gradient of high to low concentrations of ethanol in water, and washed with distilled water. The samples were stained using dyes from the HE staining kit (KGA224, KeyGEN Biotech, NanJing, China). The sections were dehydrated and dried using a gradient of low to high ethanol concentrations, and the specimens were transparent to xylene and fixed with neutral gum. Randomly selected fields of view were photographed using a phase contrast microscope (Olympus Corporation, Japan) and a 100× magnification field of view.

#### 2.4.3 Measurement of cytokine concentrations in skin tissues

Skin tissue samples were washed with pre-cooled PBS, weighed and sheared. Tissue was added to PBS at a weight-to-volume ratio of 1:9 in a glass homogenizer and ground on ice. The homogenates were centrifuged at 5000 × g for 10 min, and the supernatants decanted. The concentrations of interleukin (IL)-4 (SEKR-0004; Solarbio, Beijing, China), IL-6 (SEKR-0005; Solarbio), IL-12 (E-EL-R0064c; Elabscience, TX, USA), IL-17A (E-EL-R0566c; Elabscience), IL-23 (E-EL-R0569c; Elabscience), and interferon (IFN)-γ (E-EL-R0009, Elabscience) in skin tissues were determined by ELISA at 450 nm using a microplate reader (Berthold Technologies GmbH, Beijing, China).

#### 2.4.4 Toluidine blue staining

Wax slices of skin tissue sections were soaked in 0.5% toluidine blue solution (G1032, Servicebio, Wuhan, China) for 30 min at room temperature, washed in distilled water and immersed in 0.5% glacial acetic acid solution for 5 sec. The slices were dehydrated using a gradient of low to high concentrations of ethanol, immersed in clear xylene, cemented using neutral gum, and photographed under a light microscope (Olympus Corporation, Tokyo, Japan). Mast cells were counted in six samples per group at a magnification of ×100.

#### 2.4.5 Mast cell tryptase and eosinophil protein X assays

Immunohistochemical assays were performed using ready-to-use immunohistochemistry kits (KIT-5001, Maxim, Fuzhou, China) containing express enzyme labeled sheep anti-Mouse, rabbit, mouse/rabbit IgG, and rabbit anti-sheep IgG polymers. Paraffin sections of skin tissue samples were deparaffinized, immersed in 0.01 mol/L citrate and microwaved for 20 min to extract antigens. The sections were incubated in 0.03% H_2_O_2_ for 15 min to remove peroxidases, and incubated for 20 min at room temperature in normal goat serum blocking solution. The sections were incubated with rabbit anti-TPSAB1 (1:50 dilution; 13343-1-AP, Proteintech, Wuhan, China) or anti-EPX (1:100 dilution; bs-3881R, Bioss, Beijing, China) antibody at 4°C overnight. The sections were warmed to 37°C for 1 h and washed with phosphate buffer, followed by incubations with secondary antibody (biotin-labeled goat anti-rabbit IgG) and streptavidin for 30 min each at 37°C. The sections were stained with DAB, counterstained with hematoxylin, and sealed with neutral resin. The sections were photographed under a light microscope (Olympus Corporation, Japan) at a ×200 magnitude, and the integrated optical density (IOD) of each field of view was determined using Image-Pro Plus 6.0 software.

#### 2.4.6 Serum concentrations of IgE, leukotriene B4 and histamine

Blood samples in EP tubes were centrifuged at 3000×g for 10 min, and the serum samples removed, and frozen at -80°C (Thermo Fisher Scientific, MA, USA). The serum concentrations of IgE (SEKR-0019; Solarbio), LTB4 (CSB-E08035r; Cusabio, Wuhan, China) and HIS (E-EL-0032c; Elabscience) were determined by ELISA at 450 nm using a microplate reader (Berthold Technologies GmbH).

#### 2.4.7 Detection of autophagic vesicle number by transmission electron microscopy

Skin tissue samples were incubated in overnight at 4°C in 3% glutaraldehyde and fixed in 2% osmium tetroxide. The samples were dehydrated using an acetone gradient, embedded in epoxy resin, and cut into 70 nm ultrathin sections, which were incubated in 2% aqueous uranyl acetate, 0.8% lead citrate for 3 h at 25°C. The sections were washed in distilled water, and imaged at x12,000 using a transmission electron microscope (Thermo Fisher Scientific). The number of autophagic vesicles per unit field of view was calculated.

#### 2.4.8 Expression of Beclin-1, LC3, P62 and Ly6G in skin tissues by immunofluorescence staining

Tissue sections embedded in paraffin were immersed twice in xylene for 10 min each, dehydrated using high to low concentrations of ethanol and washed twice in double-distilled water for 5 each. The treated sections were placed in citrate buffer and microwaved for 5 min on high and 15 min on low for antigen repair. The tissue sections were shaken dry and immediately surrounded with a histochemical pen loop. The sections were incubated at room temperature for 10 min in incubated 0.1% TritonX-100, and washed three times with PBS for 5 min each. The sections were incubated in blocking solution for 30 min at room temperature, followed by incubation with primary anti-LC3 A/B antibody (1:100), anti-Beclin1 monoclonal antibody (1:50), anti-p62 polyclonal antibody (1:750), or anti-Ly6G polyclonal antibody (1:100) at 4°C overnight. The sections were washed three times with PBS. Beclin-1, LC3 and P62 were incubated with Alexa Fluor 488 labeled secondary antibody (1:200) and Ly6G were incubated with Alexa Fluor 594 labeled secondary antibody (1:200) for 1 h, and again washed three times with PBS. The plates were blocked by adding DAPI (sc-24941, Santa Cruz, CA, USA) dropwise. The sections about Beclin-1, LC3 and P62 were viewed under a laser confocal microscope, and Ly6G were viewed under a fluorescence microscope, with randomly selected fields of view photographed. Ly6G Positive cell %= Ly6G positive cell/Total cell in the field of view.

#### 2.4.9 Expression of Beclin-1, LC3, P62, LKB1, p-AMPK, AMPK, p70S6K1, p-p70S6K1, and Atg5-Atg12 complex by western blotting

Skin tissue samples were washed with pre-cooled PBS, weighed and sheared. The samples were added to RIPA buffer (P0013B, Beyotime, ShangHai, China) containing PMSF (Solon, OH, USA) at a ratio of 100 mg sample per mL buffer to lyse the cells. The samples were centrifuged at 12000 g for 5 min at 4°C, and the supernatants were transferred to pre-cooled EP tubes. Protein concentrations of the supernatants were quantified using BCA kits. An aliquot of 5×loading buffer was added to each sample, and the samples were incubated in a boiling water bath for 10 min and loaded onto SDS-PAGE gels after adding appropriate amounts of pre-cooled 1× electrophoresis buffer. The samples were electrophoresed at 80 V for about 30 min; when the bands entered the separator gel, the voltage was changed to 120 V, and the electrophoresis was performed until the target bands reached their predetermined positions. Samples were transferred to PVDF membranes (0.45 µm, Millipore, Schwalbach, Germany) that had been activated with methanol (Merch, Darmstadt, Germany) for 1 min and soaked in membrane transfer buffer for 15 min. The membranes were stained with Rexchip Red S dye for 5 min, washed twice with TBST, permeated with TBS, transferred to a sealing solution containing 5% skimmed milk powder in TBST, and sealed by shaking at room temperature for 1 h. The membrane was washed with TBST, sealed, and incubated overnight at 4°C with primary antibody to beclin-1 (1:1000; 66665-1-Ig, Prointech), LC3 A/B (1:1000; 4108, Prointech), P62 (1:5000; 18420-1-AP, Prointech), LKB1 (1:1000; 3047, Prointech), AMPK (1:1000; 5831, Proteintech), p-AMPK (1:1000), p70S6K1 (1:5000 ratio), p-p70S6K1 (1:500; ab32529, Abcam, Cambridge, UK), Atg5-Atg12 complex (1:1000; AAM79 Bio-Rad, CA, USA), or GAPDH (1:1000). The membranes were washed three times with TBST, followed by incubation at room temperature for 1 h with goat anti-rat IgG H&L (HRP; 1:10,000, ab150157, Abcam) or goat anti-rabbit IgG H&L (HRP; 1:10,000, ab6721, Abcam). The membranes were washed three times with TBST, and fully immersed in equal volumes of enhanced chemiluminescence (Thermo Fisher Scientific, Pittsburgh, PA, USA) liquid A and B solutions for 5 min. Protein expression was detected using a Tanon 6600 Luminescence Imaging Workstation and analyzed using Image Pro Plus 6.0 software. Relative protein expression was calculated as target protein gray value/inner reference protein gray value.

#### 2.4.10 Statistical analysis

Data are presented as mean ± standard deviation (SD). Multiple comparisons of normally distributed data were performed using ANOVA tests, followed by Bonferroni’s correction. Multiple comparisons data with unequal variance were performed using Welch’s ANOVA test followed by Dunnett’s T3 *post hoc* test. Multiple comparisons of abnormally distributed data were performed using the Kruskal−Wallis test followed by the Dunn’s *post hoc* test. All statistical analyses were performed using GraphPad Prism 9.0 software (GraphPad Software, Inc.), with P<0.05 considered statistically significant.

## 3 Results

### 3.1 Effect of PAL on scratching behavior

The number of scratches made by rats in the OVA group was significantly higher than that made by rats in the Control group (P < 0.001; **Figure 1A**). The numbers of scratches made by rats in the OVA+PAL and OVA+LOR groups were lower than the number made by rats in the OVA group, with the difference being statistically significant in the OVA+PAL (P < 0.05), but not in the OVA+LOR group (**Figure 1B**).

### 3.2 Effect of PAL on skin histopathology

HE staining showed that skin tissue of rats in the Control group was structurally complete and uniform in thickness. The epidermal cells and collagen fibers were uniformly stained, with complete morphology and regular arrangement (**Figure 1C**). CSU-like pathologic changes, such as capillary dilatation and inflammatory cell infiltration, were not observed. Skin tissue of rats in the OVA group was thinned and the structure was broken. Epidermal basal cells were ruptured and disorganized, and the intercellular space was visibly edematous. The collagen fiber gaps were wider and staining was lighter (blue arrow). Typical CSU-like histopathological changes, such as capillary dilatation (green arrow) and inflammatory cell infiltration (black arrow), were observed. The skin tissue structure of rats in the OVA+PAL and OVA+LOR groups was more complete. The epidermal basal cells were not obviously broken and were arranged in an orderly manner. The widening of collagen fiber gaps was mild, and the color did not become lighter. Obvious edema, capillary dilatation, inflammatory cell infiltration and other typical CSU-like histopathological changes were not observed.

### 3.3 Effect of PAL on inflammatory factors IL-4, IL-6, IL-12, IFN-γ, IL-17A, IL-23 and neutrophil recruitment

**Figure 2:**
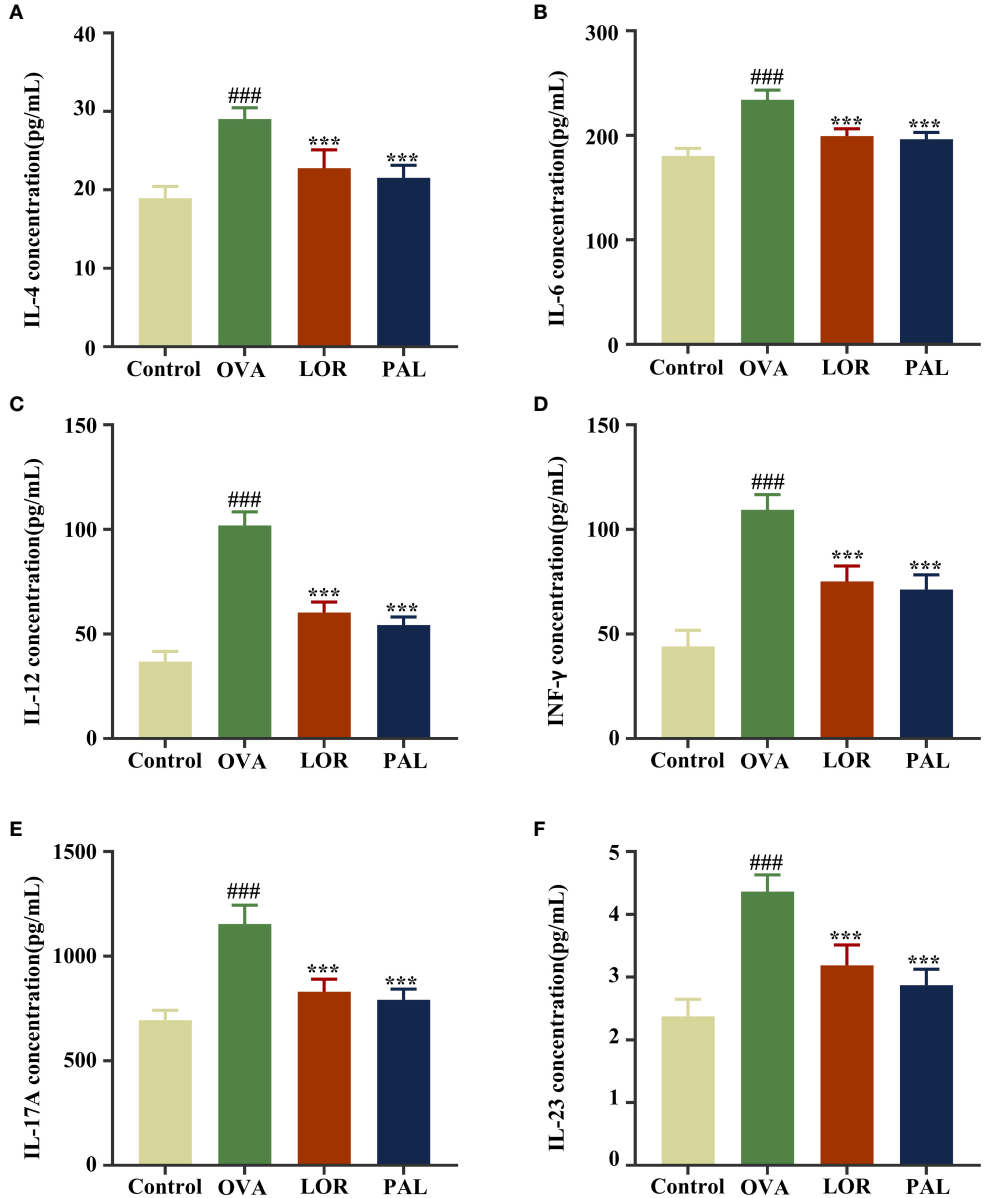
Expression of the inflammatory factors IL-4 **(A)**, IL-6 **(B)**, IL-12 **(C)**, IFN-γ **(D)**, IL-17A **(E)**, and IL-23 **(F)** in the Control, OVA, OVA+LOR and OVA+PAL groups of rats. Data are presented as mean ± standard deviation (SD). ^###^P<0.001 vs. Control group. ^***^P<0.001 vs. OVA group. OVA, ovalbumin; LOR, loratadine; PAL, palmatine; IL, interleukin; INF, interferon.

Skin concentrations of IL-4, IL-6, IL-12, IFN-γ, IL-23 and IL-17A were all significantly higher in the OVA than in the Control group (P < 0.001 each). Compared with the OVA group, however, the skin concentrations of these inflammatory factors were significantly lower in the OVA+PAL and OVA+LOR groups (P < 0.001 each; **Figure 2**).

**Figure 3:**
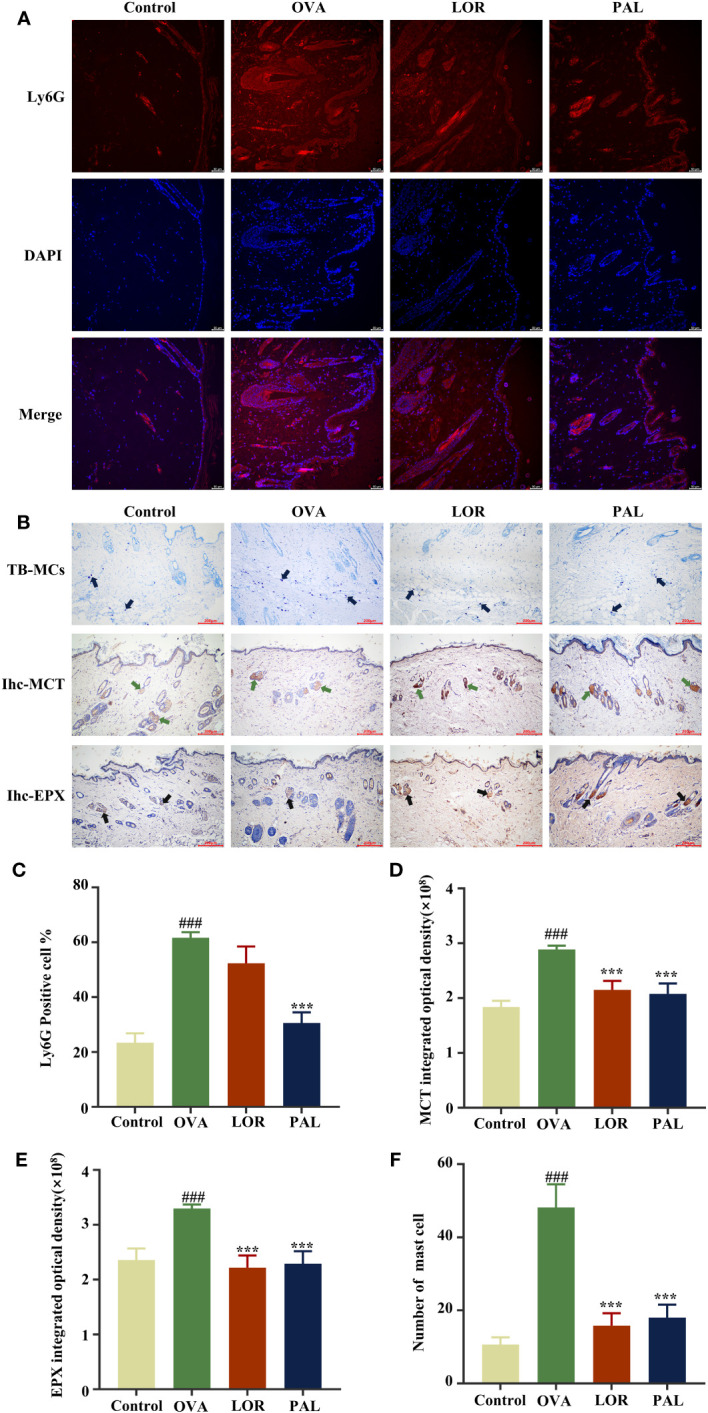
The proportion of positive cells of Ly6G **(A)**. Graphs of methylamine blue staining of mast cells and immunohistochemical staining of MCT and EXP expression in the Control, OVA, OVA+LOR and OVA+PAL groups of rats **(B)**. Fluorescence intensity of Ly6G **(C)**. Mast cell number **(D)**, MCT **(E)** and EPX **(F)** integrated optical density. Blue arrows: mast cells. Green arrows: MCT expression. Black arrows: EPX expression. Data are presented as mean ± standard deviation (SD). ^###^P<0.001 vs. Control group. ***P<0.001 vs. OVA group. OVA, ovalbumin; LOR, loratadine; PAL, palmatine; MCT, mast cell trypsin-like enzyme; MCT, mast cell trypsin; EPX, eosinophil protein X.

Compared with the Control group, the mean fluorescence intensity of Ly6G was significantly higher and neutrophil recruitment significantly greater (P < 0.001 each) in the OVA group. The proportion of positive cells of Ly6G and neutrophil recruitment were significantly lower in the LOR (P < 0.01) and PAL (P < 0.001) groups than in the OVA group (**Figures 3A, C**).

### 3.4 Effect of PAL on mast cells and EXP

The numbers of mast cells and the degranulation effect (blue arrows) were significantly higher in the OVA group than in the Control group (P < 0.001 each; **Figures 3B, D–F**), as were the IOD values of MCT (green arrows) and EXP (black arrows) (P < 0.001 each). Compared with the OVA group, the numbers of mast cells and the degranulation effect were significantly lower in the OVA+PAL and OVA+LOR groups. In addition, the IOD values of MCT and EXP were significantly lower in the OVA+PAL and OVA+LOR groups than in the OVA group (P < 0.001 each).

### 3.5 Effect of PAL on levels of allergic cytokines IgE, LTB4 and HIS

**Figure 4:**
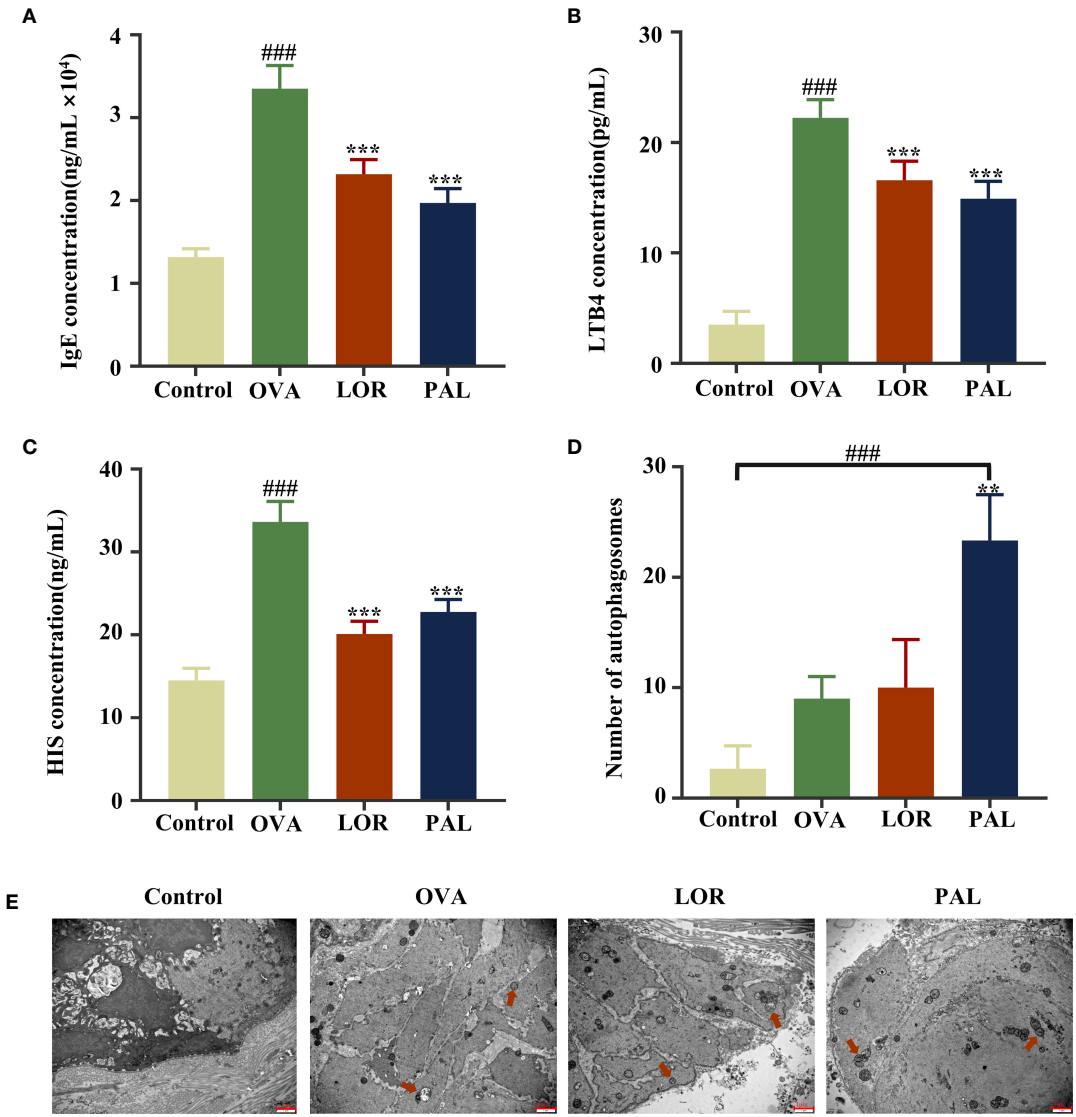
Serum concentrations of IgE **(A)**, LTB4 **(B)**, and HIS **(C)**, the numbers of autophagosomes **(D)** and autophagic activity **(E)** in the Control, OVA, OVA+LOR and OVA+PAL groups of rats. Red arrows: autophagic vesicles. Data are presented as mean ± standard deviation (SD). ^###^P<0.001 vs. Control group. ^**^P<0.01, ^***^P<0.001 vs. OVA group. OVA, ovalbumin; LOR, loratadine; PAL, palmatine; IgE, immunoglobulin E; LTB4, leukotriene 4; HIS, histamine.

The serum concentrations of IgE, LTB4, and HIS were found to be significantly higher in the OVA group than in the Control group (P < 0.001; **Figures 4A–C**). Compared with the OVA group, however, the serum concentrations of IgE, LTB4, and HIS were significantly lower in the OVA+PAL and OVA+LOR groups (P < 0.001 each).

### 3.6 Effect of PAL on autophagic activity

The epithelial cells of rats in the Control group were found to be structurally intact, with no obvious autophagic vesicles (red arrows). In contrast, the epithelial cells in the OVA group were irregularly shaped, with cytoplasmic swelling, an abnormal cellular ultrastructure, and a small number of autophagic vesicles, suggesting activation of autophagic activity. Fewer cellular abnormalities were observed in the OVA+LOR than in the OVA group, and the number of autophagic vesicles was higher in the OVA+PAL than in the OVA group, although these differences were not statistically significant. Epithelial cells in the OVA+PAL group were regularly shaped, and the abnormal cellular ultrastructure observed in the OVA group was significantly reduced. The numbers of autophagic vesicles were significantly higher in the OVA+PAL group than in the OVA and (P < 0.01) Control (P < 0.001) groups. Taken together, these results indicated that PAL could significantly enhance autophagic activity in rat epithelial cells (**Figures 4D, E**).

### 3.7 Effect of PAL on the expression of autophagy proteins

**Figure 5:**
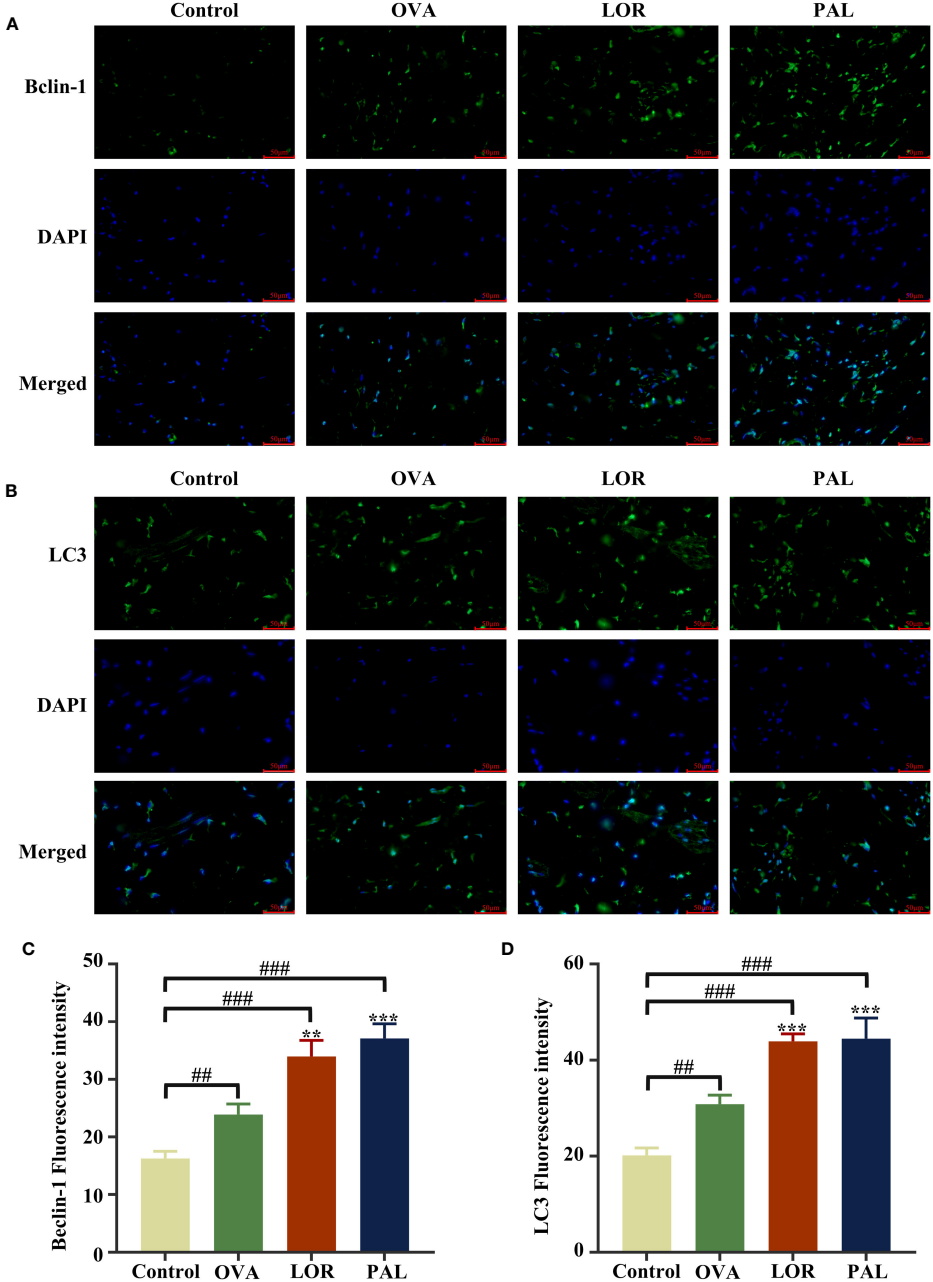
Immunofluorescence staining plots of Beclin-1 **(A)** and LC3 **(B)**, Fluorescence intensity of Beclin-1 **(C)** and LC3 **(D)**. Data are presented as mean ± standard deviation (SD). ^##^P<0.01, ^###^P<0.001 vs. Control group. ^**^P<0.01, ^***^P<0.001 vs. OVA group. OVA, ovalbumin; LOR, loratadine; PAL, palmatine.

**Figure 6:**
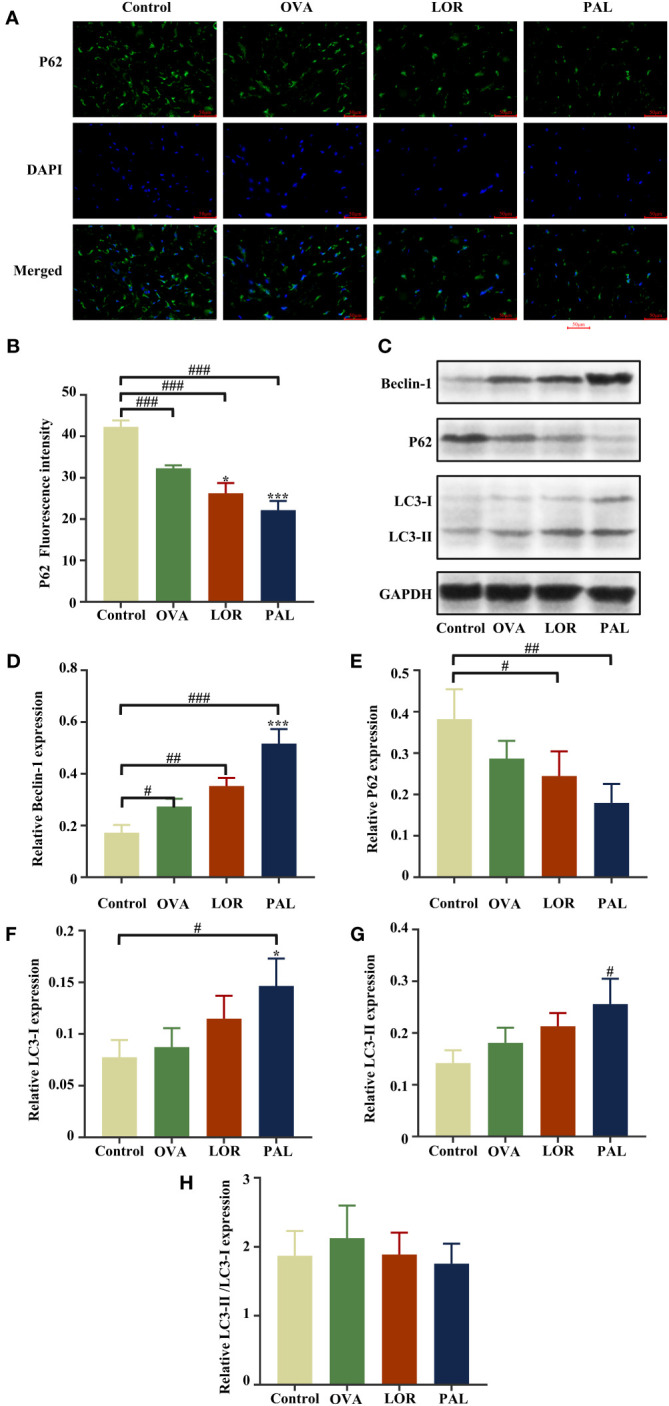
Immunofluorescence staining **(A)** and fluorescence intensity **(B)** of P62. Beclin-1, LC3-I, LC3-II, P62, and GAPDH protein bands in skin samples from the Control, OVA, OVA+LOR and OVA+PAL groups of rats **(C)**. Graphs showing the levels of expression of Beclin-1 **(D)**, P62 **(E)**, LC3-I **(F)**, and LC3-II **(G)** and LC3-II/LC3-I **(H)** in the four rat groups. Data are presented as mean ± standard deviation (SD). ^#^P<0.05, ^##^P<0.01, ^###^P<0.001 vs. Control group. ^*^P<0.05, ^***^P<0.001 vs. OVA group. OVA, ovalbumin; LOR, loratadine; PAL, palmatine.

Immunofluorescence staining of skin tissue samples showed that the levels of expression of Beclin-1 (P < 0.01) and LC3 (P < 0.001) were significantly higher, while the level of P62 (P < 0.001) was significantly lower, in the OVA than in the Control group. Compared with the OVA group, the skin of rats in the OVA+LOR group showed significant increases in the expression of Beclin-1 and LC3 (P < 0.01 reach) and a significant decrease in the expression of P62 (P < 0.05). Compared with the Control group, skin samples from rats in the OVA+LOR group showed significant increases in the expression of Beclin-1 and LC3 (P < 0.001 reach) and a significant decrease in the expression of P62 (P < 0.001). Similar to findings in the OVA+LOR group, skin samples from rats in the OVA+PAL group showed significant increases in the expression of Beclin-1 and LC3 (P < 0.001), and a significant decrease in the expression of P62 (P < 0.001) compared with the OVA group. Compared with the Control group, the skin of rats in the OVA+PAL group showed significant increases in the expression of Beclin-1 and LC3 (P < 0.001 each) and a significant decrease in the expression of P62 (P < 0.001; **Figures 5**, **6A, B**).

Western blotting also found that the level of expression of Beclin-1 was significantly higher in skin samples from the OVA than from the Control group (P < 0.05). The levels of expression of LC3-I and LC3-II were higher, and the level of expression of P62 was lower, in the OVA than in the Control group, but the differences were not statistically significant. The levels of expression of Beclin-1, LC3-I, and LC3-II were higher and the level of expression of P62 lower in skin tissues from the OVA+LOR than from the OVA group, but these differences were not statistically significant. Compared with the Control group, skin from rats in the OVA+LOR group showed a significant increase in the level of Beclin-1 (P < 0.01), non-significant increases in the expression of LC3-I and LC3-II and a significant reduction in the expression of P62 (P < 0.05). The levels of expression of Beclin-1 (P < 0.001) and LC3-I (P <0.05) in skin samples were significantly higher in the OVA+PAL than in the OVA group. In addition, the level of expression of LC3-II was higher and the expression of P62 lower in the OVA+PAL than in the OVA group, but these differences were not statistically significant. Compared with the Control group, skin from rats in the OVA+PAL group showed significantly increases in the expression of Beclin-1 (P < 0.001), LC3-I (P < 0.05), and LC3-II (P < 0.05) and a significant reduction in the expression of P62 expression (P < 0.01) (**Figures 6C–F**).

Evaluation of the expression of other autophagy proteins showed that the levels of expression of p-LKB1, p-AMPK, Atg5, Atg12 and Atg5-Atg12 complexes were significantly higher in the OVA+PAL group than in both the Control and OVA groups. In addition, the expression of p-p70S6K1 was significantly lower, in the PAL group than in the Control and OVA groups. Please refer to the **Supplementary Materials** for details of this part of figure (**Figure S1**).

## 4 Discussion

CSU is a common allergic disorder, the cause of which cannot be determined in most patients (35). Manifestations of CSU include recurring red spots and bright wheals on the head, face and body surface, and intense itching of the skin (36). These symptoms can also lead to sleep disorders, anxiety, and other adverse effects, seriously affecting a patient’s physical and mental health and significantly reducing patient quality of life (37). Scratching behavior is regarded as a behavioral indicator in UL rats to assess the relief of itching associated with CSU (38). The frequency of scratching was found to be markedly higher in UL than in Control rats, with PAL and LOR treatment found to correct this behavior and alleviate the itching symptoms in CSU. Using HE staining, the present study found that PAL and LOR treatment was associated with skin tissue changes in UL rats. Challenge with OVA alone was associated with the typical pathological changes of CSU in the skin of UL rats, such as edema and epithelial cell breakdown, as well as inflammatory cell infiltration and capillary dilatation. These pathologic, CSU-like changes in rat skin were markedly alleviated by the administration of either LOR or PAL, with PAL being more effective than LOR.

The pathogenic mechanisms of CSU were found to be closely associated with mast cell activation and eosinophilic inflammatory infiltration triggered by the high-affinity IgE (39). When antigenic substances that cause allergic reactions first enter a host, IgE binds specifically and with high affinity to mast cell surface receptors, sensitizing the host (40). Upon encountering the same antigen, the antigen will bind specifically to IgE on the surface of mast cells, inducing mast cell degranulation (41). Ultimately, this leads to CSU-like pathologic changes in skin tissue, resulting in allergic skin symptoms such as bright wheals and itching (41). MCT is an active substance that accounts for approximately 50% of the protein secreted following mast cell degranulation, with increased MCT levels often considered a biomarker of mast cell activation (42). Determination of mast cell activation by immunofluorescence staining and measurements of the levels of IgE and MCT showed that mast cell degranulation in response to activation was substantially higher in UL than in Control rats. Moreover, treatment with LOR or PAL was found to effectively attenuate mast cell activation, with PAL being more effective than LOR.

The activation of mast cells can also lead to the infiltration of eosinophils and their release of several inflammatory mediators and cytokines, which are involved in immune-inflammatory responses to CSU (43). EPX is a unique indicator of eosinophils, both because it is secreted only by eosinophils and because it the most abundant cationic protein secreted by eosinophils, suggesting that EPX may be an indicator of the extent of eosinophilic infiltration of tissues at the onset of an inflammatory response (44). Simultaneously, two powerful pro-inflammatory mediators, LTB4 and HIS, are secreted by activated mast cells to recruit inflammatory cells (45). LTB4 has been shown to activate leukocytes and eosinophils, thereby inducing vascular inflammation and tissue edema, whereas HIS has been found to alter vascular permeability and stimulate nerve endings, leading to bright wheals and itching (46).

Several common inflammatory cytokines have also been seen in CSU. IL-12 has been found to contribute to an increase in the number of Th cells, a T-cell subset that differentiates into Th1 cells; these Th cells are involved in inflammatory responses and cellular immunity, as well as in the production of several pro-inflammatory factors such as IFN-γ (47). IFN-γ plays a pro-inflammatory role in CSU by inducing the autocrine cysteine asparaginase, resulting in the differentiation of skin tissue keratinocytes, a process regarded as a form of apoptosis in skin diseases (48). IFN-γ concentrations were found to be markedly higher in patients with CSU than in the general population (49).

IL-23, a member of the IL-12 family, has been found essential for the differentiation, survival, and secretion of IL-17 by Th17 cells (50). IL-23, a constituent of the IL-23/IL-17 inflammatory axis, regulates IL-17 levels, inducing the release of pro-inflammatory mediators from basal cells, such as endothelial cells, and recruiting inflammatory cells, which can exacerbate inflammatory responses, contributing to dermal edema and then to the appearance of bright wheals (51). IL-6, a member of the IL-17 family with multidirectional pro-inflammatory effects (52), has been shown to regulate the differentiation and proliferation of T-cells, B-cells, and mast cells, and to stimulate increased synthesis of C-reactive protein and fibrinogen, all of which enhance inflammatory responses (53). IL-17 also induces the induction of chemokines that recruit neutrophils to sites of inflammation, thus exacerbating inflammatory responses (54). Using the neutrophil-specific antibody Ly6G, the present study found that PAL reduced the level of IL-17, which attenuated neutrophil recruitment to sites of inflammation. IL-6 can also induce coagulation abnormalities, the levels of which increased markedly during the acute phase of CSU (55). IL-4, another common and potent pro-inflammatory factor, is secreted by Th2 cells and induces mast cell value-added degranulation and release of inflammatory mediators by directly inducing or indirectly mediating IgE production, resulting in a vicious cycle of inflammation and exacerbating inflammatory responses (56). The present study confirmed that the levels of inflammatory mediators and pro-inflammatory cytokines are significantly higher in UL than in Control rats, suggesting that the former experience a severe inflammatory response. These responses are attenuated by LOR and PAL, with PAL having more potent activity.

Autophagy, which is strongly associated with the maintenance of environmental homeostasis in the skin, has been shown to be a potential target for the treatment of immune skin diseases (57). During attacks on the immune system, autophagy can provide relief by removing dead cells and pathogens while presenting antigens for immune recognition (58). Because autophagy is the centerpiece of innate immunity, abnormalities in autophagy usually trigger inflammatory responses that exacerbate immune disorders (59). Autophagy may play a key anti-inflammatory role by disrupting inflammatory vesicle activity and reducing the secretion of inflammatory mediators (60). Inflammatory responses triggered by Toll-like receptor 3 were found to be effectively attenuated by increasing autophagic flux in epidermal keratinocytes (61). Increasing autophagic flux by enhancing AMPK-mediated autophagic activity and decreasing P62 levels has been shown to markedly reduce allergic inflammation (62, 63). Furthermore, metformin was found to ameliorate allergic contact dermatitis by activating autophagy to suppress macrophage activation and NOD-like receptor pyrin structural domain protein 3 inflammatory vesicle activity (64). Activation of autophagy can also alleviate imiquimod-induced psoriasiform dermatitis (65). Autophagy-related proteins are highly expressed in epidermal cells, where they play a therapeutic role following the occurrence of psoriatic inflammatory responses (66). Jing Fang granules have been found to improve autophagic flux by modulating LKB1/AMPK/SIRT1, thereby alleviating glucose metabolism disorders and inflammatory responses in mice with CSU (67). Although less is known about autophagy in CSU, its critical importance remains evident.

Autophagy enhances normal cell survival by lysosomally degrading damaged cells and their contents, thereby maintaining environmental homeostasis and energy levels (68). The autophagic process can be divided into three main steps: activation of autophagy, autophagosome formation, and fusion degradation (69). LKB1 is a major kinase that induces AMPK phosphorylation. Activation of the LKB1/AMPK pathway has been found to effectively attenuate IgE-induced mast cell degranulation, an important step in allergic diseases (70). High AMPK levels have an anti-inflammatory effect, reducing the release of inflammatory mediators (71). High expression of AMPK will also reduce the level of the downstream factor mTOR, thus promoting autophagy (63). The level of phosphorylated p70S6K1, which acts as a downstream signal for mTOR to mediate the inhibition of autophagy, is regarded as a marker of mTOR activity (72). The present study found that the expression of LKB1 with phosphorylated AMPK was significantly elevated, whereas the level of phosphorylated p70S6K1 was significantly decreased, in UL rats. Taken together, these findings suggest that autophagy was initiated at the onset of UL.

These trends were strengthened following treatment with PAL and LOR, confirming that PAL enhances the activation of autophagy in UL rats. Beclin-1 has been found to regulate autophagy and to form complexes, which induce autophagosome production and maturation, mainly from the emergence of autophagic activity to its peak (73). Beclin-1 is therefore an essential factor in the formation of autophagosomes during autophagy (74). The present study found that beclin-1 expression was higher in OVA+PAL than in OVA UL rats, suggesting that PAL could significantly enhance autophagosome formation.

Members of the Atg family are key proteins in the autophagy fusion chain. A lysine residue in Atg5 is often linked to the C-terminal glycine residue of Atg12, formomg a ubiquitin-like conjugation system (75). This Atg5-Atg12 complex can help autophagic vesicles undergo membrane elongation, while directly localizing on the outer membrane of autophagic vesicles and determining the degree of vesicle curvature to facilitate the next step of fusion (76). The Atg5-Atg12 complex also allows LC3 to pool toward autophagosomes and can induce the fusion of autophagosomes and lysosomes (77). LC3 exists in two forms, cytoplasmic LC3-I and membrane LC3-II, with the key to autophagosomal lipid-membrane fusion being the formation of membrane-lipidated LC3-II after the aminocoupling of cytoplasmic LC3-I with phosphatidylethanolamine (78). LC3-I and LC3-II are mainly responsible for the extension of the autophagosome membrane, allowing further expansion of autophagosome volume (79). LC3-II acts together with P62, the “garbage truck” in autophagy, to recognize and wrap autophagic substrates, to continuously concentrate and aggregate these substrates, to transport them to autophagic vesicles, and finally to bind them to lysosomes for degradation (80). The present study showed that the levels of expression of Atg5-Atg12 complex and LC3-II were significantly higher, and P62 were markedly lower in the OVA+PAL and OVA+LOR groups than in the OVA group, suggesting that the autophagy fusion degradation process was significantly activated. Calculation of LC3-II/LC3-I ratios showed that LC3-I expression was elevated in the OVA, OVA+PAL and OVA+LOR groups, resulting in a decrease in their LC3-II/LC3-I ratios. This may be explained as the PAL induces the accumulation of LC3 I, which provides more substrate for the conversion to LC3 II. Because the transition from LC3-I to LC3-II is a dynamic process, total amounts may not always be consistent (81, 82). Based on its transformation process, we speculate that it may be due to the surge in autophagy function that causes autophagosomes and lysosomes to fuse at speed, during which LC3-II on the outer membrane is cleaved to produce excessive LC3-I, with LC3-II on the inner membrane being degraded by lysosomal enzymes, reducing the LC3-II/LC3-I ratio (83). It is not sufficiently convincing to show that autophagy flux is altered by “LC3II/Loading Control protein” alone (84). Therefore, we combined the results of several autophagy-related proteins to analyze the results. It was observed that the OVA attack resulted in an increase in the expression levels of LC3, Beclin-1, p-LKB1, p-AMPK, Atg5, Atg12, and Atg5-Atg12, while P62 and p-p70S6K1 expression decreased. Furthermore, the administration of PAL and LOR is expected to further intensify these observed trends. These findings indicated that autophagy had been activated in UL rats after OVA challenge, but to a low degree. Following treatment with PAL or LOR, the activities of various steps in autophagy increased to counteract the deleterious effects of OVA. Moreover, PAL showed a greater ability to activate autophagic flux than LOR.

In summary, the present study showed that PAL can effectively alleviate itch and other CSU-like pathological changes in UL rats, through a mechanism of action associated with the reduction of inflammatory responses and increases in autophagic activity. PAL was more potent than LOR, a drug clinically used to treat CSU. These results provide a definite pharmacodynamic basis for further research and development of PAL. It is worth noting that more and more studies mention that type I autoallergy and type IIb autoimmunity will coexist in the pathogenesis of CSU (85). Therefore, we will plan to conduct relevant studies on the role of PAL intervention on type II.b autoimmunity.

## Data availability statement

The original contributions presented in the study are included in the article/**Supplementary Material**. Further inquiries can be directed to the corresponding author.

## Ethics statement

The animal study was approved by Professional Committee for animal ethics of Yunnan University of Chinese Medicine (Approval No. R-062022157). The study was conducted in accordance with the local legislation and institutional requirements.

## Author contributions

TX: Data curation, Software, Writing – original draft. XZY: Writing – original draft, Validation. LPY: Project administration, Writing – review and editing. XHD: Project administration, Writing – review and editing, Funding acquisition, Resources.
