# Supplementary material for: Palmatine treats urticaria by reducing inflammation and increasing autophagy
Source: Front Immunol. 2023 Nov 14;14:1268467. doi: 10.3389/fimmu.2023.1268467 (PMC10682667; doi:10.3389/fimmu.2023.1268467)

Supplementary Material

Palmatine treats urticaria by reducing inflammation and increasing autophagy

**TIAN XIAO, XINGZHI YU, LIPING YANG and XIAOHUA DUAN***

**^*^Correspondence:**

Dr Xiao-Hua Duan, Yunnan Key Laboratory of Dai and Yi Medicine, Yunnan University of Chinese Medicine, 1076 Yuhua Road, Chenggong, Kunming, Yunnan 650500, P.R. China

E-mail: 1047896527@qq.com

# Supplementary Data

## Effect of PAL on the expression of autophagy proteins p70S6K1, LKB1, AMPK, Atg5, Atg12

As shown by the western blotting results, compared with the Control group, the level of p-LKB1 in rats in the OVA group was obviously increased (P < 0.01), and the expression of p- AMPK, Atg5, Atg12, and Atg5-Atg12 complex was elevated and the expression of p-p70S6K1 was decreased, but the difference was not statistically significant. After the LOR intervention in rats, p-AMPK was markedly elevated in skin tissues compared with the OVA group (P < 0.01), and the expression of p-LKB1, Atg5, Atg12, and Atg5-Atg12 complex was elevated and p-p70S6K1 expression was decreased, but the difference was not statistically significant. Compared with the Control group, the rats in the LOR group had markedly elevated p-LKB1 and p- AMPK (P < 0.001, P < 0.01), but not statistically significant differences in Atg5, Atg12, and Atg5-Atg12 complex, and p-p70S6K1 expression (P < 0.01). The expression of p-LKB1 and p- AMPK was extremely elevated in the PAL group of rats compared with that of the OVA group (P < 0.05, P < 0.001). The expression of Atg5, Atg12, and Atg5-Atg12 complex was increased but the difference was not statistically meaningful, and the expression of p-p70S6K1 was obviously decreased (P < 0.05). Compared with the Control group, the expression of p-LKB1 and p- AMPK was extremely elevated in rats in the PAL group (P < 0.001). The expression of Atg5, Atg12, and Atg5-Atg12 complex was significantly increased (P < 0.05), and the expression of p-p70S6K1 was obviously decreased (P < 0.01). (Figure 7)

# Supplementary Figures and Tables

Figure S1. (A) Western blotting results, showing protein bands in skin samples from the Control, OVA, OVA+LOR and OVA+PAL groups of rats. (B–J) Graphs showing the levels of expression of p70S6K1 (B), p-p70S6K1 (C), LKB1 (D), p-LKB1 (E), AMPK (F), p-AMPK (G), Atg5 (H), Atg12 (I), and Atg5-Atg12 complex (J) in these rats. Data are presented as mean ± standard deviation (SD). #P<0.05, ##P<0.01, ###P<0.001 vs. Control group. *P<0.05, **P<0.01, ***P<0.001 vs. OA group. Abbreviations: OVA, ovalbumin; LOR, loratadine; PAL, palmatine.


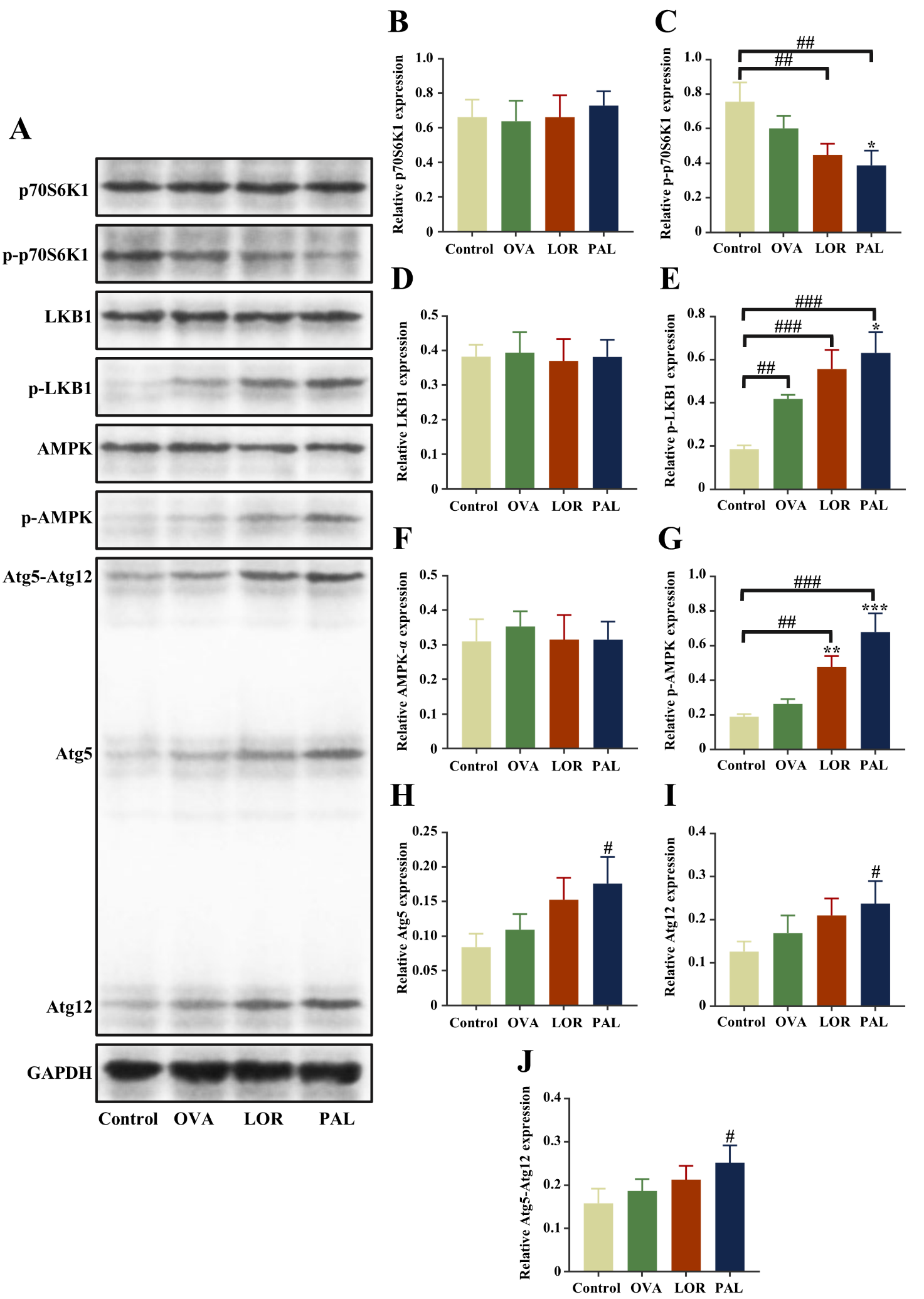

Supplement: Supplementary file 1 [file DataSheet_1.docx]
